# Ethical dilemmas in prioritizing patients for scarce radiotherapy resources

**DOI:** 10.1186/s12910-024-01005-3

**Published:** 2024-01-31

**Authors:** Rebecca J. DeBoer, Anita Ho, Espérance Mutoniwase, Cam Nguyen, Grace Umutesi, Jean Bosco Bigirimana, Nicaise Nsabimana, Katherine Van Loon, Lawrence N. Shulman, Scott A. Triedman, Vincent K. Cubaka, Cyprien Shyirambere

**Affiliations:** 1 Division of Hematology/Oncology, University of California, San Francisco, San Francisco, CA USA; 2 University of British Columbia, Vancouver, British Columbia, Canada; 3 University of Rochester, Rochester, NY USA; 4 University of Colorado Cancer Center, Aurora, CO USA; 5 University of Washington, Seattle, Washington, USA; 6 Partners in Health/Inshuti Mu Buzima, Kigali, Rwanda; 7 University of Pennsylvania Abramson Cancer Center, Philadelphia, PA USA; 8 Warren Alpert Medical School of Brown University, Providence, Rhode Island, USA

**Keywords:** Health Care Rationing, Resource Allocation, Priority setting, Radiotherapy, Global Health, Africa, Rwanda

## Abstract

**Background:**

Radiotherapy is an essential component of cancer treatment, yet many countries do not have adequate capacity to serve all patients who would benefit from it. Allocation systems are needed to guide patient prioritization for radiotherapy in resource-limited contexts. These systems should be informed by allocation principles deemed relevant to stakeholders. This study explores the ethical dilemmas and views of decision-makers engaged in real-world prioritization of scarce radiotherapy resources at a cancer center in Rwanda in order to identify relevant principles.

**Methods:**

Semi-structured interviews were conducted with a purposive sample of 22 oncology clinicians, program leaders, and clinical advisors. Interviews explored the factors considered by decision-makers when prioritizing patients for radiotherapy. The framework method of thematic analysis was used to characterize these factors. Bioethical analysis was then applied to determine their underlying normative principles.

**Results:**

Participants considered both clinical and non-clinical factors relevant to patient prioritization for radiotherapy. They widely agreed that disease curability should be the primary overarching driver of prioritization, with the goal of saving the most lives. However, they described tension between curability and competing factors including age, palliative benefit, and waiting time. They were divided about the role that non-clinical factors such as social value should play, and agreed that poverty should not be a barrier.

**Conclusions:**

Multiple competing principles create tension with the agreed upon overarching goal of maximizing lives saved, including another utilitarian approach of maximizing *life-years* saved as well as non-utilitarian principles, such as egalitarianism, prioritarianism, and deontology. Clinical guidelines for patient prioritization for radiotherapy can combine multiple principles into a single allocation system to a significant extent. However, conflicting views about the role that social factors should play, and the dynamic nature of resource availability, highlight the need for ongoing work to evaluate and refine priority setting systems based on stakeholder views.

**Supplementary Information:**

The online version contains supplementary material available at 10.1186/s12910-024-01005-3.

## Background

Low- and middle-income countries (LMICs) face an unprecedented growth in cancer burden and disproportionate share of global cancer deaths [1]. For the most common cancers in LMICs, radiotherapy is an essential component of effective treatment [2]. However, there is a severe shortage of radiotherapy resources worldwide, including equipment and personnel, and many African countries lack adequate capacity to serve their populations [3]. In 2020, radiotherapy was available in only half of African countries, and operational radiotherapy capacity covered less than 10% of cancer cases requiring radiotherapy in Eastern, Central, and Western Africa [4]. This mismatch between supply and demand inherently means that some patients in need do receive radiotherapy while others do not, creating a need for priority setting.

Priority setting, an umbrella term that encompasses both healthcare rationing and resource allocation, occurs at all levels of the healthcare system [5]. At the level of patient care, decisions about individual patients’ access to limited resources are referred to as microallocation, or “bedside rationing.” Generations of ethicists have articulated theories and principles to guide microallocation of scarce healthcare resources such as solid organs, intensive care beds, and vaccines in a pandemic (i.e., allocation principles) [6]. Policymakers and practitioners have operationalized these principles into explicit allocation systems. Ideally, allocation systems are informed by empirical research on stakeholder views in order to establish legitimacy [7]. For example, researchers have investigated the public’s views on hypothetical healthcare rationing dilemmas [8–10] and patients’ and clinicians’ views on actual healthcare rationing dilemmas [11–14]. In the absence of explicit priority setting, resources tend to be distributed implicitly based on morally arbitrary or ad hoc determinants, such as ability to pay, social privilege, or a first-come, first-served basis.

Despite the number of overburdened radiotherapy machines globally, there are no widely accepted systems for patient prioritization across heterogeneous clinical indications in low resource settings. Frameworks have been proposed to guide radiotherapy resource allocation at the macro healthcare system level in LMICs, [15, 16] and a handful of microallocation systems from high income countries (HICs) with universal coverage and long waiting lines have been published [17–20]. However, these microallocation systems are not readily transferrable to low resource contexts, where supply-demand mismatch may not be merely a matter of waiting times but rather of access to any radiotherapy at all. Moreover, the allocation principles deemed relevant for priority setting may differ across diverse cultural and sociopolitical contexts, and few empirical studies on bedside rationing have been conducted in Africa [21–24]. 

Given that priority setting is central to everyday clinical decisions in low resource settings, there is both a need for further research to understand the allocation principles deemed relevant to decision makers in these contexts, and an opportunity to learn from their real-world expertise. This study aims to explore the ethical dilemmas and factors considered in patient prioritization for scarce radiotherapy resources at a cancer center in rural Rwanda, and to examine the normative implications of participants’ views. Our immediate objective was to inform local clinical guidelines and procedures for radiotherapy microallocation. Our broader objective was to develop an empirical account of the principles deemed relevant to decision makers engaged in real-world healthcare microallocation in Rwanda.

## Methods

### Setting

Butaro Hospital is a district hospital in rural Rwanda run by the Ministry of Health (MOH) and supported by the social justice non-governmental organization Partners In Health (PIH), known locally as Inshuti Mu Buzima (IMB). In 2012, the first cancer treatment center in Rwanda was established at Butaro Hospital through international partnership, with a mission to deliver high quality cancer care for poor and rural populations [25]. The Butaro Cancer Center of Excellence (BCCOE) provides basic services across the cancer care continuum, including pathologic diagnosis, surgery, chemotherapy, palliative care, and psychosocial support. For specialized services that are not available on site at BCCOE, including radiotherapy, externals referrals are provided when possible. With no oncology specialists permanently on site, oncology care has been delivered by local and international internists, pediatricians, a general surgeon, general practitioners, and nurses in routine consultation with U.S.-based clinical advisors through a task shifting model [26]. The clinical advisors are medical and radiation oncologists based at U.S. academic partner institutions who provide clinical advice, teaching, and mentorship to BCCOE clinicians during weekly virtual tumor board conferences and regular in-person visits.

Until 2019, there was no radiotherapy in Rwanda, and public resources for patients to be treated outside the country were very limited. At BCCOE, PIH/IMB was able to financially support a limited number of patients per month to receive radiotherapy in neighboring countries. From 2012 to 2016, cohorts of BCCOE patients traveled to the Uganda Cancer Institute (UCI) by bus every one to two months to receive radiotherapy. In 2016 the radiotherapy machine at UCI broke down beyond repair, and thereafter smaller cohorts of BCCOE patients were flown to Nairobi Hospital in Kenya for radiotherapy. Initially, funds were adequate to send most patients who required radiotherapy for curative treatment. As patient volumes grew and the need for radiotherapy began to outstrip supply, patients were added to a waiting list, creating the obligation for BCCOE physicians to prioritize patients. At the time, there were no pertinent institutional or national guidelines for the microallocation of scarce resources. The team developed clinical guidelines for patient prioritization that were designed to maximize curative benefits of radiotherapy, and established regular selection meetings to facilitate group rather than individual decision-making [27]. Patients who might have benefitted from radiotherapy for palliation of pain or for urgent management of complications were unable to be sent due to both financial and logistical constraints, and were treated with other modalities if indicated or with palliative care.

Access to radiotherapy for BCCOE patients expanded substantially in 2019 with the installation of a radiotherapy unit at Rwanda Military Hospital in Kigali. However, despite this major leap forward, demand continues to exceed supply. Coverage through public insurance or PIH/IMB sponsorship remains constrained. Staffing shortages, machine breakdowns, and maintenance issues result in bottlenecks. Thus, priority setting will be required for the foreseeable future to optimize resource utilization.

### Study design and participants

We conducted a qualitative interview study to understand the experiences and views of those engaged in radiotherapy priority setting and patient care at BCCOE to inform future guidelines and procedures for patient prioritization. Purposive sampling was used to recruit current and former physicians and nurses who have provided oncology care at BCCOE (collectively, “clinicians”), program leaders, and U.S.-based clinical advisors. Participants were recruited onsite at BCCOE through verbal invitation or offsite by email. The study was led by a former physician now clinical advisor and researcher at BCCOE (R.D.) and the BCCOE Director of Oncology who is also an oncology clinician (C.S). To protect anonymity, here we refer to Rwandan and other East African participants as “Local” and participants from outside East Africa as “Non-Local.”

This article focuses on the normative principles considered in patient prioritization for radiotherapy at BCCOE. Separate analyses of procedural fairness [27] and of the moral distress and resilience experienced by clinicians engaged in cancer care priority setting [28] are reported elsewhere.

This study was approved by the Rwanda National Ethics Committee, the Inshuti Mu Buzima Research Committee, and the University of California San Francisco Institutional Review Board.

### Data collection

An interview guide was developed by a multidisciplinary team of study investigators with expertise in global oncology, bioethics, qualitative research methods, and the radiotherapy referral program at BCCOE. Pertinent to this article, questions explored participants’ views about the mission of BCCOE and the factors that do, and should, determine patient prioritization for radiotherapy (see Supplemental File). Initial open-ended questions were followed by probes asking participants to evaluate whether and how palliative benefit, social value, age, gender, and socioeconomic status should be considered. Semi-structured interviews were conducted by R.D. All participants provided written informed consent. After the first two interviews, the guide was revised to enhance clarity and flow. Interviews were audio-recorded and transcribed verbatim. Transcripts were de-identified to protect confidentiality.

### Data analysis

The framework method of thematic analysis was used to analyze the textual data [29]. This method was developed for applied qualitative research and uses a structured approach to inform pre-determined objectives. The framework method was chosen for this study because we planned to apply our qualitative data directly to the improvement of procedures for radiotherapy prioritization at BCCOE. An analytical framework was developed through a combination of conceptual categories in the interview guide and those that emerged inductively during an initial open coding phase. All transcripts were independently coded by R.D. and a co-investigator (E.M. or C.N.), and A.H. performed an independent consistency check. Intercoder agreement was assessed and discussed by all coders for each transcript, and discrepancies were adjudicated by consensus. The analytical framework was continually refined during the coding process. Matrices for each conceptual category were created in spreadsheets, with columns for each theme and rows for each participant. Textual data were then charted into the matrices. Data were summarized and interpreted globally and by column. MAXQDA (VERBI Software; Berlin, Germany) was used for data management and analysis.

## Results

Characteristics of the 22 participants are presented in Table 1.

**Table 1:** Participant characteristics (*N* = 22)

Participant Characteristics	N	%
**Gender**		
Female	7	32%
Male	15	68%
**Role(s) at Butaro (not mutually exclusive)**		
Oncology Nurse	3	14%*
Oncology Physician	13	59%*
Program Leader	7	32%*
Clinical Advisor	4	18%*
**Role Status at Time of Interview**		
Former	5	23%
Current	17	77%
**Nationality**		
Rwandan	9	41%
American	9	41%
Other	4	18%
**Local vs. Non-Local Classification**		
Local (East African)	11	50%
Non-Local (from outside East Africa)	11	50%
**Interview Characteristics**		
In-person	14	64%
*Mean duration in minutes (range)*	*52*	*(32—91)*
Telephone	8	36%
*Mean duration in minutes (range)*	*46*	*(25—62)*

*Categories are not mutually exclusive; percentages do not add up to 100%

Participants identified both clinical and non-clinical factors that were considered in patient prioritization for radiotherapy at BCCOE. Maximizing opportunities for cancer “cure,” and thus saving lives, was widely perceived to be the foremost goal of the radiotherapy program. Accordingly, disease curability and clinical factors that affect the likelihood of cure were upheld as the appropriate primary determinants of prioritization. However, participants described tension between curability and competing factors including age, palliative benefit, and waiting time. They were divided about the role that non-clinical factors such as social value should play, and agreed that poverty should not be a barrier.

### Curability

Participants unanimously affirmed that curability has been, and should be, the primary overarching driver of patient prioritization for radiotherapy in the setting of limited resources. In general, patients were only considered eligible for radiotherapy if their disease was potentially curable *and* radiotherapy was required for cure. Within this designation, patients were prioritized based on their estimated chance of cure. Thus, clinical features that determine curability, such as cancer type and stage, were frequently referenced. As a representative example:

In my mind, a high chance of curability always trumps other things. (P08)

From a programmatic perspective, participants framed curative benefit in terms of maximal return on investment, or getting the “biggest bang for the buck” (P13). Rwandan participants tended to also link cure to societal benefit, noting that the “social impact of our money” is greater if a patient is cured than if survival is prolonged for a couple of years (P09), and asserting that “the few resources available should be used effectively to serve the people who are likely to be cured and to resume to society.” (P01).

### Curability versus age

Given that there are more potentially curable patients than available resources, most participants believed that potential *life-years* gained by curing disease (as a function of age) should also be considered. Many illustrated the interaction between curability and age through examples of a clinical scenario requiring a choice between an older versus younger patient with similar chances of cure:

You have somebody who is 90 years old, you have somebody who is 20 years old; the overall benefit of curing the 20-year-old is going to be more substantial since they have many more years of life ahead of them. And I think that scales down in a valid way. It’s a tough choice to make, but if you have a stage Ib cervical cancer patient who is 20 and one who is 50 or 60, and you can only send one of them, I don’t think it’s unreasonable to send the younger person. (P12)

Others illustrated the tension between curability and age through examples of having to choose between an older patient with a higher chance of cure and a younger patient with a lower chance of cure. They invoked additional factors related to age, such as the opportunity to live a normal lifespan. For instance, a local program leader explained that Rwanda’s average life expectancy of 65 [sic] provided justification for selecting a younger patient over one in her 50s who might live a nearly average lifespan with palliative chemotherapy alone. (P09) A local physician ascribed the value in non-curative survival prolongation at a younger age to providing the opportunity to live through more of life’s stages:

A 45-year-old with cervical cancer who’s had children, married, and has lived—yes, she’ll benefit more, she’ll be cured—but there’s this child who could get 5 more years…” (P07).

The concept of life-years was especially salient to clinicians in choosing between adult and pediatric candidates. Participants reported that in reality, children with a curative indication for radiotherapy are typically sent, even if the incremental survival benefit of radiotherapy may be less than for adults on the waiting list:

[Age is] the number one thing we hang our hat on, that final push all the time. “C’mon, she’s young. She has many good years ahead of her. Give her a chance to live.” Even more in ped[iatric]s—obviously, we don’t even debate peds cases. We just give them X spots when they need them. And I don’t think anyone would disagree with that. We really feel that amount of life—or just the chance to live—is worth something. (P20)

Moreover, favoring children over adult patients, regardless of the likelihood of cure, was perceived by physicians to be acceptable to the local community, including to the adult patients who might be affected. (P02)

### Curative versus palliative benefit

Despite widespread agreement that curable patients should be prioritized, participants expressed concerns of not being able to send patients for palliative radiotherapy, which is often indicated for pain relief, urgent management of complications such as cord compression or bleeding, or disease control in the non-curative setting:

We have a huge gap… All patients who have an indication, even for palliative [radio]therapy, should go to have this treatment. It’s palliative but it helps them to have a better life before they die. But we are not able to offer this opportunity, not because we don’t want to, but because we are limited in terms of resources. (P05)

Several suggested that tumor burden and the magnitude of potential palliation should be considered, referencing disfiguring and functionally limiting facial tumors or foul-smelling fungating masses that are not only painful but associated with stigma and risk of abandonment by a spouse, family, or community. They invoked principles such as the human right to pain control and to a dignified death in emphasizing that all patients *should* be able to receive palliative radiotherapy:

“Even if you have to die, you have to die in dignity.” (P06).

When pressed, however, participants unanimously affirmed that it would be unacceptable for a curable patient to lose their chance of cure because a patient with incurable disease was prioritized for a spot. As a local nurse explained, if you send a patient for palliative radiotherapy instead of a curable patient, that curable patient will also progress to an incurable stage, so “you are losing two patients, [when] you could lose one and save the other one.” (P04).

Participants across categories acknowledged the moral tension between clinicians’ role as stewards of a scarce resource and their professional obligation to treat the individual patient in front of them, which was especially pronounced in the face of an incurable patient who could derive substantial palliative benefit from radiotherapy. As one physician explained:

I have to think as a clinician who wants to improve [care] for my patient, but also as an economist who has to use effectively the resources we have. (P01)

Yet advisors discussed the flaws of a dichotomous view of curative and palliative intent, asserting that the priority placed on curative benefit should depend on more nuanced factors such as the *likelihood* of cure, pace of disease, and risk of toxicity. For example, while a patient with a 90% chance of cure should clearly take precedence over a patient with a palliative indication, one with a 5% chance of cure might not. (P08) Or, patients who have incurable but indolent disease and could live for many years with radiotherapy should potentially be prioritized over those with a modest chance of cure. Advisors also emphasized that curability should be weighed against the morbidity and risks of radiotherapy, particularly in light of concerns about technical capacity and availability of ancillary care at partner radiotherapy facilities. (P13) For example, for pediatric indications such as nephroblastoma,

The risks [of radiotherapy] are huge, and the toxicity is high, and the chance of cure is there but relatively small compared to the risks. Just because there’s a chance of cure doesn’t mean that they should be prioritized. (P11)

Several participants also noted the availability of morphine and other pain medications at Butaro as alternative palliation strategies, contextualizing prioritization decisions within the scope of treatment options.

### Curability versus waiting time

Several clinicians and advisors discussed the daunting clinical and moral challenge posed by the waiting list. Because the guidelines prioritized curability, new cases of early-stage cancer were routinely chosen over patients on the waiting list with later stages, and as patients waited, their disease progressed further.

For example, you have a patient who is cervical cancer stage IIB, in category one [highest priority]. She cannot go now. Next time you meet, she has progressed to IIIB. And you have another eight patients who are stage IIB. The tendency is to keep sending these IIBs, while this person has been [waiting]. So, the decision is, do I send IIIB who has a low chance of cure, or do I ignore this person and keep sending the IIBs? That has been a very tough discussion, and sometimes we have to close our eyes and say, we cannot send many IIIBs even though they have been on the list. Just give them a few spots. (P18)

Some participants advocated for the opposite approach of prioritizing patients with later stages who are closer to losing their window for cure. For example:

There [are] patients who are curable, but if it’s not done as soon as possible, they will end up being metastatic and non-curable. And you see on the list, they are low priority, while practically-speaking, we can save them. [For] those people, curable but advanced stage, if there is a way to make them go to radiotherapy as soon as possible, that [should be] a modification. … For example, a cervical patient IB can wait 3 months. But a nasopharyngeal carcinoma IVA, in 1 month he can metastasize. (P01)

### Social value

Participants were more divided about the role of non-clinical patient factors in prioritization decision-making. Half of participants reported that social value does, or should, play a role in patient selection for radiotherapy. Some candidly confessed to allowing social value and health behaviors to affect decision-making, as did this local physician:

I’ll be honest. If a patient—for example, single man, heavy drinker, heavy smoker, early stage or has a chance of cure, but has those habits… I would choose a mother with 5 children, less chance of cure, over him. So that has come up. (P07)

Others upheld this stance more unapologetically, as with this program leader:

I don’t think [social value] *can’t* come into play. They’re moral judgements just like age is to some extent. It might be arbitrary, and they may seem relatively clean cut at some points… In the best of all worlds, you treat everybody who could potentially benefit, but that’s not where we are, and you’re going to have to use some criteria to choose however many patients a month. If it’s two 30-year olds, and one is a drug addict and in prison all the time and the other one is a mother of 3 young children who is subsistence farming, it’s hard not to take those factors into consideration. Whether it’s ethically fair or not, I don’t know, but if we had those two patients sitting in a room and I could only send one of them, I know who I’d send. (P12)

One local participant ascribed consideration of social value to good leadership:

If you are going to choose, as an institution [with] good leadership, you could think, what’s the benefit? The benefit is that you will have a patient who will come back and do some beneficial activities for the community. She was a mother, she needed to take care of kids, a family, her husband. So that could be [considered], if I’m a good leader. (P04)

As in these examples, participants consistently illustrated the role of social value through hypothetical scenarios in which a mother of young children is selected over an older person. When asked specifically, some denied that gender is or should be an independent factor in selection, while others acknowledged a gender bias explicitly due to expected social responsibilities:

People are more sensitive to women. It’s not objective, it’s not written anywhere, but if I consider those social conditions, who is going to take care of kids, most of the time I will select to save a woman’s life. (P06)

In contrast, the other half of participants were firmly opposed to considering a patient’s value to society when selecting for radiotherapy. They alluded to the equal worth of all human lives, both implicitly and explicitly, when asked if social value should affect prioritization:

Even though you are useless in the community or you are causing harm, we just say, we are [going] to treat you; even though you are a drug addict… we treat everyone. (P02)

Others emphasized the methodological difficulties with considering social value, i.e., it is impossible to measure and not stable over time, as well as the hazards: “that’s so fraught with potential for abuse” (P08) and “there is so much bias and prejudice that goes into an assessment of social value.” (P17) A local program leader asserted that it would be impossible to operationalize social value due to its subjectivity:

We have never considered [social value]. And we are not planning to consider that because we look at a patient as a patient, not his role in the community. Because if you consider that, the next time you can say, oh this one is a teacher, he’s teaching a class of fifty people; this other one is just a casual farmer, if she dies… You can never bring that because it can be very subjective—how do you judge who is more important in the community than the other? (P18)

### Ability to pay

Participants agreed that resources should be used for patients who cannot otherwise afford radiotherapy, referencing PIH’s core value of providing a preferential option for the poor. Since the vast majority of patients at BCCOE fall into this category, socioeconomic status was generally not considered in patient selection. However, participants expressed differing views about patients who fall in a “gray area” of affordability. For example, a local program leader suggested that patients who are prominent community members, such as a church leader, may be able to raise funds, which would reserve resources for poor patients without a social network. (P09) Conversely, others warned of the consequences of asking patients and families to use their savings and community resources to pay for radiotherapy, noting that the most common cause of personal bankruptcy in the United States is having cancer. (P12) One physician explained that ability to pay becomes more relevant with lower priority clinical indications for radiotherapy, while pointing out that determining ability to pay is methodologically challenging. Others noted that poverty can pose significant barriers to potential radiotherapy candidates even if financial costs are covered by PIH. For example, navigating travel logistics and making childcare arrangements may be daunting for patients without resources. (P08)

## Discussion

This study presents an empirical account of the ethical dilemmas and views of clinicians, program leaders, and advisors engaged in routine microallocation of scarce radiotherapy resources at a cancer center in rural Rwanda. Participants widely agreed that curability should be the primary driver of patient prioritization for radiotherapy. However, tension arises when curability conflicts with other factors including age, palliative benefit, and waiting time. They were divided about whether social value should be considered. The factors that participants believe should (or should not) determine patient prioritization constitute moral judgments that reflect underlying allocation principles (Table 2). It is useful to understand complex views about priority setting in terms of normative principles in order to incorporate them into real world policies and procedures [8]. The leading approach to establishing legitimacy and fairness in healthcare priority setting, Accountability for Reasonableness (AFR), requires that decisions appeal to the principles deemed most relevant to stakeholders [30]. As we previously reported, our participants placed greater importance on this substantive “relevance” condition of AFR than the procedural conditions (i.e., transparency, revisability, and enforcement) [27]. In this discussion we apply bioethical analysis to identify the principles behind the clinical and non-clinical factors that decision-makers deem morally relevant when prioritizing patients, enabling us to optimize the legitimacy and fairness of radiotherapy priority setting in Rwanda.

**Table 2:** Participant Views and Underlying Principles for Resource Allocation

Participant Views	Relevant Allocation Principle
Curative benefit should drive radiotherapy prioritization	Utilitarian goal of saving the most lives
Life expectancy after cure should be considered	Utilitarian goal of saving the most life-years
Children should be given a chance to live more life stages	Prioritarian goal of favoring “youngest first”
Duty to alleviate immediate suffering with palliative radiotherapy	Moral and professional duty (deontology); Prioritarian goal of favoring “sickest first” or Rule of Rescue
Contributors to society (e.g. mothers) should be prioritized	Instrumental social value
High risk behaviors (e.g. tobacco use) should be penalized	Social value and reciprocity
Impoverished patients should be prioritized	Social justice

Participants expressed consensus that prioritization should be driven primarily by the goal of maximizing opportunities for “cure,” or lives saved, reflecting the utilitarian principle of maximizing total benefits. Saving lives is the ultimate mission of the MOH and PIH, and overall survival is the gold standard measure of benefit in oncology. Resource-stratified clinical practice guidelines for LMICs and the World Health Organization Essential Medicines List also determine the value of cancer interventions based on survival benefits, affirming their preeminence in global oncology [31–33]. Other benefits, such as quality of life and toxicity avoidance, are incorporated as well, but survival is ubiquitously prioritized [34]. Thus, an emphasis on curability as the appropriate primary determinant of radiotherapy allocation is consistent with local and international values.

Yet participants also endorsed consideration of life expectancy after radiotherapy, reflecting another utilitarian goal: saving the most *life-years* [35]. Using prognosis as a measure of benefit is supported by precedent in global oncology as well. For example, the global burden of cancer has been estimated in terms of disability-adjusted life-years (DALYs) by the Global Burden of Disease Study [36]. The principle of maximizing life-years, generally operationalized at BCCOE as prioritizing younger over older patients with similar chances of cure, was broadly acceptable to our participants. Several participants linked maximizing life-years to a societal benefit, reminiscent of applying DALYs to evaluate the impact of health on economic development [37]. Under the development paradigm, health interventions are seen as means for achieving economic growth through maximizing DALYs and thus productivity, not as ends per se [38]. Our participants’ references to “return on investment” reflect this value framework.

However, tension arises when maximizing life-years conflicts with maximizing lives saved. In some cases—and consistently in pediatric cases—patients with greater life expectancy are prioritized for radiotherapy even if their chance of cure might be lower than older patients. Participants justified prioritizing the young over the old based on non-utilitarian rationales as well. Some supported prioritizing patients whose age is lower than Rwanda’s average life expectancy, reflecting the principle of equal opportunity to live a normal lifespan [39]. Others invoked the life-cycle (or “fair innings”) principle to defend prioritizing children who have indications for radiotherapy regardless of the magnitude of curative benefit. This stance values the opportunity afforded by life-prolonging radiotherapy to experience more of life’s stages, even if it is not curative [40]. A “youngest first” approach of prioritizing those who would otherwise die having lived the fewest life stages is also a form of prioritarianism, or favoring the worst off [41]. Clinicians and local program leaders regarded the practice of always prioritizing children with potentially curable disease as “obvious,” even though it was not necessarily aligned with advisor recommendations and it deviated from the BCCOE clinical guidelines, which are based on incremental survival benefit. Various empiric surveys of people’s views on healthcare priority setting support prioritizing the young over the old [40]. All of these justifications for prioritizing patients based on age pose tension with the overarching principle of maximizing lives saved.

As with curability versus age, the conflict between curative versus palliative intent created significant moral tension. This conflict resembles the classic conflict between utilitarianism, which focuses on the consequences of actions, and deontology, which focuses on ethical duty and the rightness or wrongness of actions independent of their outcomes [5]. Participants—especially clinicians—emphasized their duty to treat the individual patient in front of them in service of an established therapeutic relationship. They frequently see patients who could derive significant palliative benefit from radiotherapy, for example to alleviate painful bone metastases or foul-smelling fungating tumors, and feel a moral and professional duty as clinicians to provide this symptom relief. Additional principles support this view, such as a “sickest first” interpretation of prioritarianism, or the related “Rule of Rescue” intuition to alleviate identifiable, avoidable suffering [42]. Many participants also referenced a human right to pain relief [43]. However, when pressed, they unanimously upheld the decision to prioritize saving a curable patient over palliating an incurable patient, viewing the practice of not sending patients for palliative radiotherapy as a tragic but necessary consequence of scarcity. They expressed optimism that as radiotherapy access expands in Rwanda, resources will be made available to cover the costs of palliative radiotherapy as well.

Several participants described tension between curability and time spent on the radiotherapy waiting list, expressing uncertainty about how to address the dimension of time when managing the list. Some conveyed an intuition to prioritize those who had been waiting longer, again appealing to a moral and professional duty to individual patients within a therapeutic relationship, and to a sense of fairness as defined by treating people similarly. Others advocated for prioritizing patients who are on the verge of becoming incurable (e.g., stage IIIB cervical cancer) over patients with early-stage disease who may still be cured even if their treatment were delayed. This stance reflects an underlying prioritarian approach of favoring the worst off, or those with the worst future prospects if left untreated [44]. As participants acknowledged, both of these practices would conflict with the overriding goal of maximizing survival benefits. This dilemma highlights that the dynamic nature of curability challenges a utilitarian system that is based on chance of cure at a given timepoint.

Participants were divided about whether social value should be considered in prioritization decisions. Broad social value refers to one’s overall worth to society, involving summary judgments about past and future contributions to society’s goals [35]. Several participants believed that patients who contribute to society, typically through taking care of dependents, should be prioritized, and it would be acceptable to deprioritize those who they thought were draining societal resources. Some also considered it appropriate to reward healthy lifestyle choices, or conversely, to deprioritize individuals whose behaviors, such as tobacco or alcohol use, might have contributed to their cancer risk. In contrast, participants who strongly opposed consideration of social value or behaviors argued that it was not only wrong to judge the moral worth of others’ lives, but impossible to operationalize, and rife with potential for bias and abuse. Following the consideration of social value in allocating scarce dialysis machines in the 1960s in the United States, there has been widespread rejection of the idea that one individual is more worthy of saving than another, reflecting an egalitarian view [35]. Nevertheless, public surveys have supported prioritizing those who take care of young children, or those who have avoided behaviors such as smoking, drug abuse, and heavy drinking [40]. 

Finally, our participants unanimously agreed that poverty should not be a barrier to radiotherapy access, reflecting an egalitarian view that people with equal needs should benefit equally, regardless of socioeconomic status. Many went further to suggest that the poorest should be prioritized over those with more means, reflecting the principle of social justice [45]. Some participants endorsed consideration of ability to pay in rare cases of patients who can afford radiotherapy privately, especially for lower priority indications. In the theoretical literature, ability to pay is not regarded as a plausible option for allocating scarce life-saving interventions [6]. Nevertheless, healthcare distribution *is* based on ability to pay in many parts of the world, reflecting the impacts of capitalism, neoliberalism, and libertarianism.

Thus, significant pluralism—and, often, tension—are revealed by applying bioethics frameworks to glean normative principles from our participants’ views. Despite consensus that maximizing curative benefit should generally drive radiotherapy prioritization, this principle frequently conflicts with other non-utilitarian principles deemed highly relevant. AFR does not provide specific procedural guidance for resolving conflict between relevant rationales; thus, other approaches are needed [46]. A common approach is to incorporate morally relevant principles into multiprinciple allocation systems [6]. For example, the United Network for Organ Sharing (UNOS) systems for organ allocation combine the principles of sickest-first, prognosis, and first-come, first-served, weighting principles differently depending on the organ distributed. A prominent ventilator allocation system for public health emergencies such as the COVID-19 pandemic combines saving the most lives, saving the most life-years, and the life cycle principle [35]. Combining principles into allocation systems increases complexity and also controversy, since people may disagree about how to balance different principles, but is necessary to incorporate multifaceted moral perspectives into a unified approach. Some of our participants suggested developing a UNOS-like system to address the multiple factors considered relevant to radiotherapy prioritization at BCCOE, acknowledging that a rigorous effort to do this would be complicated and resource intensive [27]. 

The clinical guidelines for radiotherapy prioritization at BCCOE provide a potential opportunity for integrating different principles into one system. These guidelines operationalize the principle of maximizing curative benefit by ranking categories of cancer type and stage by the incremental overall survival benefit conferred by radiotherapy for each category based on available data in the oncology literature [47]. Participants confirmed that the guidelines are heavily relied upon for patient selection at BCCOE, noting their role in promoting objectivity and mitigating moral distress [27, 28]. While the guidelines are primarily based on the survival benefits of radiotherapy, they have the potential to incorporate other principles. For instance, pediatric indications are given highest priority in the revised guidelines, regardless of the incremental curative benefit of radiotherapy for these indications, based on our findings that the principles of maximizing life-years saved and favoring the young over the old take precedence for our participants. Thus, the guidelines arbitrate the tension between the overarching utilitarian emphasis on saving lives and the intuition to give children a chance of cure, which is supported by other principles. Ongoing research is needed to evaluate and continually refine the clinical guidelines in order to optimally incorporate relevant principles.

### Limitations

Several limitations should be considered when interpreting this study. As a qualitative study in a purposive sample of BCCOE decision-makers, the views expressed here may not represent all stakeholders at BCCOE or at other institutions in Rwanda. Moreover, the perspectives of patients and of the Rwandan public were not included. BCCOE is a unique collaboration between a government, a non-governmental organization, and international academic partners at a rural district hospital, without on-site clinical radiation oncology expertise, which may not be generalizable to other contexts. Nevertheless, we believe that this context, in which priority setting dilemmas are explicit and deliberate efforts have been made to address these dilemmas through a lens of social justice, offers a valuable opportunity to understand the allocation principles that are important to decision makers in real-world situations. While qualitative studies of particular contexts are inherently limited in generalizability, their strength lies in providing an in-depth understanding of complex processes and interacting factors. Future research should elicit the views and prioritization practices of professionals in other resource-constrained settings and of a broader group of stakeholders, potentially including patients and the general public, across diverse contexts.

## Conclusions

An examination of the allocation principles that decision makers apply to ethical dilemmas in radiotherapy prioritization in Rwanda demonstrates that multiple competing principles conflict with the agreed upon overarching goal of maximizing lives saved. These competing principles include an alternative utilitarian approach of maximizing *life-years* saved as well as non-utilitarian principles, such as egalitarianism, prioritarianism, and deontology. To a significant extent, clinical guidelines for patient prioritization for radiotherapy can combine multiple principles into a single allocation system. However, conflicting views about the role that social factors should play highlight the need for further deliberation. Moreover, the dynamic nature of radiotherapy resource availability and expansion in Rwanda and elsewhere underscores the need for ongoing work to evaluate and refine priority setting systems to respond to current circumstances and views. This work is resource-intensive itself, a paradox that calls for innovative and context-appropriate strategies. Our study can serve as a model for incorporating the underrepresented perspectives of decision makers in resource-limited contexts into priority setting systems through analysis of underlying normative principles.

### Electronic supplementary material

Below is the link to the electronic supplementary material.

Supplementary Material 1

## Data Availability

Data may be made available upon request and with permission from the Inshuti Mu Buzima Research Committee.

## Abbreviations

AFR: Accountability for Reasonableness
BCCOE: Butaro Cancer Center of Excellence
DALYs: Disability-adjusted life-years
HICs: High income countries
LMICs: Low- and middle-income countries
MOH: Ministry of Health
PIH/IMB: Partners In Health/Inshuti Mu Buzima
UCI: Uganda Cancer Institute
UNOS: United Network for Organ Sharing
