# Supplementary material for: Ethical dilemmas in prioritizing patients for scarce radiotherapy resources
Source: BMC Med Ethics. 2024 Jan 31;25:12. doi: 10.1186/s12910-024-01005-3 (PMC10829165; doi:10.1186/s12910-024-01005-3)
Supplement: Supplementary file 1 — Supplementary Material 1 [file 12910_2024_1005_MOESM1_ESM.docx]

**INTERVIEW GUIDE**

**Study Aim:** To understand the qualitative experience and views of oncology clinicians, nurses, and program leaders engaged in radiotherapy allocation decision-making at Butaro Hospital in Rwanda.

**INTRODUCTION**

Thank you for agreeing to an interview today, I greatly appreciate you taking the time to meet with me.

The aim of this study is to understand the experience and views of providers and program leaders who are engaged in decision-making about radiotherapy (RT) allocation at Butaro Hospital. This study is part of a larger project that we hope will improve this decision-making process at Butaro and ultimately be disseminated to other similar settings. To meet this goal, we believe it is extremely important to hear directly from stakeholders like yourself who have experience with RT allocation decisions.

I am interested in hearing your stories, thoughts, and suggestions about RT allocation decision-making. Please feel free to share your honest opinions. There are no right or wrong answers. Your views and suggestions are very valuable. We will use what we learn in these interviews to approve the process for RT allocation decision-making at Butaro.

I have a list of questions I would like to ask you. Feel free to bring up any topics you feel are related to our discussion.

Also, I would like to remind you that your participation in this interview is entirely voluntary. I recognize that discussing experience with resource allocation can be uncomfortable or distressing. You may refuse to answer any question or choose to stop participating at any time.

I would like to record our discussion so that I do not miss anything you say. Our discussion will remain completely anonymous and confidential. Your name will not be used and any identifying information will be removed. The information you give will only be used for the purpose answering this research question.

Do you agree to participate in the interview?

The interview will last about 30 to 45 minutes. Do you have any questions before we begin?

**ONCOLOGY PROGRAM AT BUTARO HOSPITAL**

1. In your own words, what is the overall mission (goal / purpose) of the oncology program at Butaro?
2. How would you describe the scope of cancer treatment at Butaro?
   1. What treatment options are available?
   2. What types of cancer are treated at Butaro?
   3. What types of cancer are *not* treated at Butaro?
3. What is your understanding of how decisions have been made about which cancers to treat versus not to treat at Butaro?
   1. …which treatment options to provide versus not to provide? (i.e., which chemotherapy regimens)
   2. Who was/is involved in this decision-making process?
   3. What factors are considered when deciding which cancers to treat / treatments to provide?
4. To your knowledge, how has the scope of cancer treatment at Butaro changed since the program first started (if at all)?

**RADIOTHERAPY REFERRAL SYSTEM AT BUTARO**

1. Please describe your understanding of how the radiotherapy (RT) referral system at Butaro works.
   1. (If applicable) Could you describe how the RT referral system changed or evolved from the time you started at Butaro? i.e., “Tell the story” of the RT program at Butaro
2. How does the RT referral system work in real-life compared to how it is supposed to work ideally?
3. In your view, how does the RT referral program fit within the mission of the oncology program?

**VALUES IN RADIOTHERAPY ALLOCATION DECISION-MAKING**

1. In your opinion, what do you think are the most important factors to consider in prioritizing patients for limited RT resources, and why?
   1. Patient level and program level
2. In our discussion so far, you’ve mentioned several factors and values that are considered in selecting patients for RT, such as [e.g., disease, stage, age, etc.] I am going to read a list of [other] values that could potentially be considered in making prioritization decisions; please tell me your thoughts about which of these are more or less relevant.
3. Clinical benefit:
   1. Survival benefit (Chance of cure)
   2. Palliative benefit (Pain control)
4. Social value
5. Age
6. Gender
7. Nationality
8. Ability to pay
9. Time on waiting list

**PERSONAL EXPERIENCE WITH RADIOTHERAPY ALLOCATION DECISIONS**

1. How has the experience of being involved in decisions about allocating limited resources for RT affected you (i.e., emotionally, professionally)?

**PROCESS IN RADIOTHERAPY ALLOCATION DECISION-MAKING**

1. How do you think RT allocation decisions *should* be made at Butaro?
2. What is the ideal decision-making process?
3. What do you think should be done differently?
4. If there are conflicting opinions about prioritization, how should they be resolved?
5. Which stakeholders should be involved in setting priorities for RT allocation?
6. Should patients or patient advocates be involved?
   - 1. If yes: how might they best be involved?
     2. If no: why not?
7. Do you believe patients and the public have access to the RT prioritization guidelines? (Why or why not?)

**CLOSING**

1. Do you have any ideas or practical suggestions to improve RT allocation at Butaro?

We are now coming to the end of the interview. Is there anything else you would like to add?

Thank you again so much for taking the time to talk with me today, I appreciate hearing about your experiences.
